# Neonatal lesions of orbital frontal areas 11/13 in monkeys alter goal-directed behavior but spare fear conditioning and safety signal learning

**DOI:** 10.3389/fnins.2014.00037

**Published:** 2014-03-04

**Authors:** Andy M. Kazama, Michael Davis, Jocelyne Bachevalier

**Affiliations:** Yerkes National Primate Research Center and Department of Psychology, Emory UniversityAtlanta, GA, USA

**Keywords:** orbitofrontal cortex (OFC), flexible decision-making, safety-signal processing, non-human primate development, areas 11 and 13

## Abstract

Recent studies in monkeys have demonstrated that damage to the lateral subfields of orbital frontal cortex (OFC areas 11/13) yields profound changes in flexible modulation of goal-directed behaviors and deficits in fear regulation. Yet, little consideration has been placed on its role in emotional and social development throughout life. The current study investigated the effects of neonatal lesions of the OFC on the flexible modulation of goal-directed behaviors and fear responses in monkeys. Infant monkeys received neonatal lesions of OFC areas 11/13 or sham-lesions during the first post-natal week. Modulation of goal-directed behaviors was measured with a devaluation task at 3–4 and 6–7 years. Modulation of fear reactivity by safety signals was assessed with the AX+/BX− fear-potentiated-startle paradigm at 6–7 years. Similar to adult-onset OFC lesions, selective neonatal lesions of OFC areas 11/13 yielded a failure to modulate behavioral responses guided by changes in reward value, but spared the ability to modulate fear responses in the presence of safety signals. These results suggest that these areas play a critical role in the development of behavioral adaptation during goal-directed behaviors, but not or less so, in the development of the ability to process emotionally salient stimuli and to modulate emotional reactivity using environmental contexts, which could be supported by other OFC subfields, such as the most ventromedial subfields (i.e., areas 14/25). Given similar impaired decision-making abilities and spared modulation of fear after both neonatal lesions of either OFC areas 11 and 13 or amygdala (Kazama et al., 2012; Kazama and Bachevalier, 2013), the present results suggest that interactions between these two neural structures play a critical role in the development of behavioral adaptation; an ability essential for the self-regulation of emotion and behavior that assures the maintenance of successful social relationships.

## Introduction

The ability to process and flexibly respond to quickly changing social information requires the complex interaction between many brain areas, including the components of the orbitofronto-limbic circuit. Great strides have been made in elucidating the role of each of the structural nodes within this circuit through an array of neuroscience tools, including neuroimaging, neurophysiology, and behavioral lesion studies. For example, cross-talk between the amygdala and the orbital frontal cortex is known to be critical for using cost-benefit information to guide optimal decision-making (see Murray and Wise, 2010, for review). This conclusion is based on several tract-tracing studies demonstrating strong bidirectional connections between the amygdala and the orbital frontal cortex in the non-human primate brain (see Barbas, 2000; Ongur and Price, 2000 for review). Moreover, interruption of connections between these two neural structures using cross-disconnection lesions (Baxter et al., 2000), in which unilateral lesions of the two regions in contralateral hemispheres are combined with section of the commissures, profoundly altered the abilities of nonhuman primates to avoid responding for stimuli that predicted a devalued reward. Disruption of orbitofrontal-amygdala cross talks during development has been associated with poor decision making skills frequently reported in several neuropsychiatric disorders, such as Post-Traumatic Stress Disorder (PTSD; Shin et al., 2006), anxiety disorders (Del Casale et al., 2012), schizophrenia (Shepherd et al., 2012), and Autism Spectrum Disorder (ASD; Barbaro and Dissanayake, 2007; Reed et al., 2013). Thus, there is a growing need to better define the critical role of the orbital frontal cortex and amygdala in the ability to make appropriate decisions and to flexibly regulate behavior during development.

To fulfil this goal, our approach was to evaluate the effects of selective damage to either the amygdala or OFC areas 11 and 13 in infant monkeys using a variety of behavioral and cognitive tasks across development (Bachevalier et al., 2011; Kazama and Bachevalier, 2012; Kazama et al., 2012; Raper et al., 2013). In recent publications, we showed that neonatal amygdala lesions impaired the ability to modulate animals' defensive responses toward different social signals depicted by a human intruder's gaze direction and this deficit emerged in infancy and persisted throughout adulthood (Raper et al., 2012). These same animals with neonatal amygdala lesions failed to update choice preferences when the rewarding value of stimuli was changed (Kazama and Bachevalier, 2013). Yet, despite a slight retardation in fear conditioning, animals with neonatal amygdala lesions discriminated normally between cues signaling fear and cues signaling safety and, more remarkably, were able to use safety cues to regulate their reactivity to the fear cues as did the control animals (Kazama et al., 2012). As discussed in an earlier report (Kazama and Bachevalier, 2013), these differential effects of neonatal amygdala lesions on social and rewarding cues vs. fear conditioning suggest that the amygdala may rely on the rapid updating (on the span of a single exposure) of the valence of external or internal cues to guide optimal decision making and emotional reactivity; a function that may likely be realized by the functional interactions between the amygdala and orbital frontal cortex. If this proposal is correct, it is likely that a similar dichotomy may be found when the neonatal lesions are restricted to the orbital frontal cortex. To test this possibility, the current series of experiments assessed the effects of selective neonatal lesions of orbital frontal areas 11 and 13 on the development of flexible decision-making abilities, using two translational tasks. Experiment 1 utilized the Reinforcer Devaluation paradigm previously employed in humans (O'Doherty et al., 2001; Gottfried et al., 2003), rodents (Colwill and Rescorla, 1985; Pickens et al., 2003; Zeeb and Winstanley, 2013), and monkeys (Malkova et al., 1997; Baxter et al., 2000; Machado and Bachevalier, 2007a; West et al., 2012) to measure behavioral adaptation to changes in reward value. Experiment 2 utilized the AX+/BX− fear-potentiated startle paradigm, similarly employed across humans (Jovanovic et al., 2012), rodents (Myers and Davis, 2004), and monkeys (Winslow et al., 2008) to assess condition inhibition. The results demonstrate that, as for the neonatal amygdala lesions, the neonatal orbital frontal lesions altered the abilities to flexibly shift object choices away from those items associated with devalued food reward while sparing fear conditioning, safety signal learning, conditioned inhibition, and extinction. A summary of preliminary findings have been previously published in either reviews (Bachevalier et al., 2011; Jovanovic et al., 2012) or abstracts (Kazama et al., 2008, 2010).

## Materials and methods

### Subjects

Ten rhesus macaques (*Macaca mulatta*) of both sexes (4.5–8 kg) participated in this study at approximately 3–4 and 5–6 years of age for the reinforcer devaluation task, which was directly followed by the AX+/BX− Fear-potentiated startle paradigm. Animals had received operations between 8 and 12 days of age, which included either aspiration lesions of areas 11 and 13 of the orbitofrontal cortex (Group Neo-Oasp, 2 males, 3 females) or sham-operations (Group Neo-C, 2 males, 3 females). However, due to behavioral issues, only four animals in Group Neo-C participated in Experiments 1 and 2 (see Tables 2, 4 for individual cases). All procedures were approved by the Animal Care and Use Committees of the University of Texas Health Science Center at Houston and of Emory University. As the descriptions of both the rearing conditions as well as lesion extents have appeared in previous publications (Goursaud and Bachevalier, 2007; Bachevalier et al., 2011; Jovanovic et al., 2012; Kazama and Bachevalier, 2012), only a summary is provided below.

As newborns, animals were individually housed, and maintained on a 12 h light/dark cycle. In addition to daily contact with peers, animals were also given daily contact with human caregivers. At 1 year of age, four animals were housed in larger cages to allow permanent social contact with peers. Animals were fed age-appropriate diets and water was provided *ad-libitum*.

Monkeys received several behavioral tests prior to the studies as well as between the two ages at which the reinforcer devaluation task was given. The tasks included measuring recognition/relational memory abilities (Bachevalier, unpublished data), object discrimination reversal learning (Kazama and Bachevalier, 2012), emotional reactivity to fearful stimuli (Raper et al., 2013), social attachment (Goursaud and Bachevalier, 2007), and peer social interactions (Payne et al., 2007).

### Surgical procedures

All procedures have already been described in details in earlier reports (Goursaud and Bachevalier, 2007; Kazama and Bachevalier, 2012). Both control and experimental groups received Magnetic Resonance Imaging-guided surgical procedures performed according to strict adherence to ethical and safety guidelines as provided by NIH and the University of Texas-Houston Institutional Animal Care and Use Committee. The pre-surgical brain imaging included a 3D T1-weighted fast spoiled gradient (FSPGR)-echo sequence (*TE* = 2.6 ms, *TR* = 10.2 ms, 25° flip angle, contiguous 1 mm sections, 12 cm FOV, 256 × 256 matrix) obtained in the coronal plan that was used to precisely visualize the position of the orbital frontal sulci serving as landmarks for the surgical removal of areas 11 and 13 (Machado and Bachevalier, 2006; Machado et al., 2009).

Following the MRI scans, animals were kept anesthetized in the stereotaxic apparatus and brought immediately to the surgical suite where they were prepared for the surgical procedures that were performed under aseptic conditions. For the sham-operations, a small craniotomy was performed in both hemispheres just in front of bregma and the dura was then cut, but no aspiration lesions were performed. For the orbital frontal cortex lesion, the bone was opened as a crescent just above each supra-orbital ridge to gain access to the orbital frontal surface. With the aid of a surgical microscope and the use of small 21 and 23 gauge aspirating probes, cortical areas 11 and 13 of the orbital frontal cortex were gently aspirated. The anterior border of the lesions were a line joining the anterior tip of the lateral and medial orbital sulci, and the posterior border ended at the location where the olfactory striae begun to turn laterally. Laterally, the lesion ended at the medial lip of the lateral orbital sulcus and, medially, at the lateral border of the stria olfactory. Within these borders, the lesion included most of areas 11 and 13 and a small anterior portion of Ia (anterior insula) posteriorly.

After the surgical procedures, the wound was sutured in anatomical layers, the animals were then removed from the Isoflurane gas anesthesia and allowed to recover in an incubator ventilated with oxygen. Treatments were started 12 h before surgery and continued until post-surgical day 7. All monkeys received both pre and post-surgical antibiotic treatments (Cephazolin, 25 mg/kg, per os) to reduce the chance of infection as well as dexamethazone sodium phosphate (0.4 mg/kg, s.c.) to control post-surgical swelling. Additionally, a topical antibiotic ointment/anesthetic was applied to the wound each day and Acetaminophen (10 mg/kg, p.o.) was administered four times a day for 3 days after surgery to relieve pain and hasten recovery.

### Lesion verification

Post-surgical *in vivo* neuroimaging investigation of the extent of the neonatal orbital lesions has already been described in details in several reports (Goursaud and Bachevalier, 2007; Kazama and Bachevalier, 2012) and estimation of the lesion extent is given for each case in Table 1. In the present paper, we present postmortem histological investigation of the lesion extent.

**Table 1:** **Extent of intended and unintended damage in Group Neo-Oasp**.

**Cases**	**Areas 11 and 13**				**Area 10**				**Area 12**			
	**L**	**R**	**Avg**	**W**	**L**	**R**	**Avg**	**W**	**L**	**R**	**Avg**	**W**
Neo-Oasp-1	86.8	83.1	85.0	71.6	0	0	0	0	40.2	11.0	25.6	4.4
Neo-Oasp-2	81.0	97.8	89.4	79.6	5.3	0	2.6	0	9.3	1.4	5.4	0.1
Neo-Oasp-3	96.4	91.2	93.8	88.0	7.4	12.3	9.8	0.9	22.3	21.6	22.0	4.8
Neo-Oasp-4	85.7	94.8	90.2	81.2	0	0	0	0	2.8	4.0	3.4	0.1
Neo-Oasp-5	90.4	98.0	94.3	88.6	6.2	10.2	8.2	0.6	18.5	22.8	20.6	4.2
X	88.1	93.0	90.5	81.8	3.78	4.5	4.1	0.3	18.6	12.2	15.4	2.7
**Cases**	**Area 14**				**Ia**				**Area 46**			
	**L**	**R**	**Avg**	**W**	**L**	**R**	**Avg**	**W**	**L**	**R**	**Avg**	**W**
Neo-Oasp-1	8.0	10.2	9.1	0.8	11.6	3.4	7.5	0.4	0	0	0	0
Neo-Oasp-2	31.9	6.8	19.4	2.2	78.5	57.7	68.1	45.3	0	0	0	0
Neo-Oasp-3	18.7	11.6	15.1	2.2	16.5	13.8	15.1	2.3	0	0	0	0
Neo-Oasp-4	9.7	12.6	11.2	1.2	82.5	64.6	73.6	53.3	0	0	0	0
Neo-Oasp-5	6.5	11.0	8.5	0.7	87.0	67.8	77.4	59.0	0	0	0	0
X	15.0	10.4	12.7	1.4	55.2	41.5	48.3	32.1	0	0	0	0

*Data are the estimated percentage of damage as assessed from MR (post-surgical T1) images. L, percentage of damage to the left hemisphere; R, percentage of damage to the right hemisphere; Avg, average of L and R; W = (L × R)/100 [weighted index as defined by Hodos and Bobko (1984)]; X, group mean. Areas 10, 11, 12, 13, 14, and 46, cytoarchitectonic subregions of the macaque frontal lobe and Ia, agranular insular areas as defined by Carmichael and Price (1994)*.

**Figure 1:**
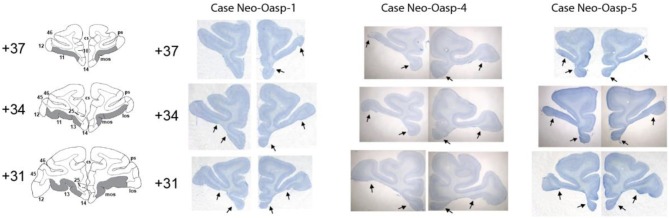
**Intended damage is shown in gray on coronal sections through the orbital frontal cortex of an infant macaque brain atlas in the left column**. Matched thionin-stained sections are provided in the right-hand column for three cases (Neo-Oasp-1, -4, and -5). Arrows point to borders of lesions for each coronal level. Abbreviations: mos, medial orbital sulcus; los, lateral orbital sulcus; numbers refer to Brodmann areas (Brodmann, 1909).

**Figure 2:**
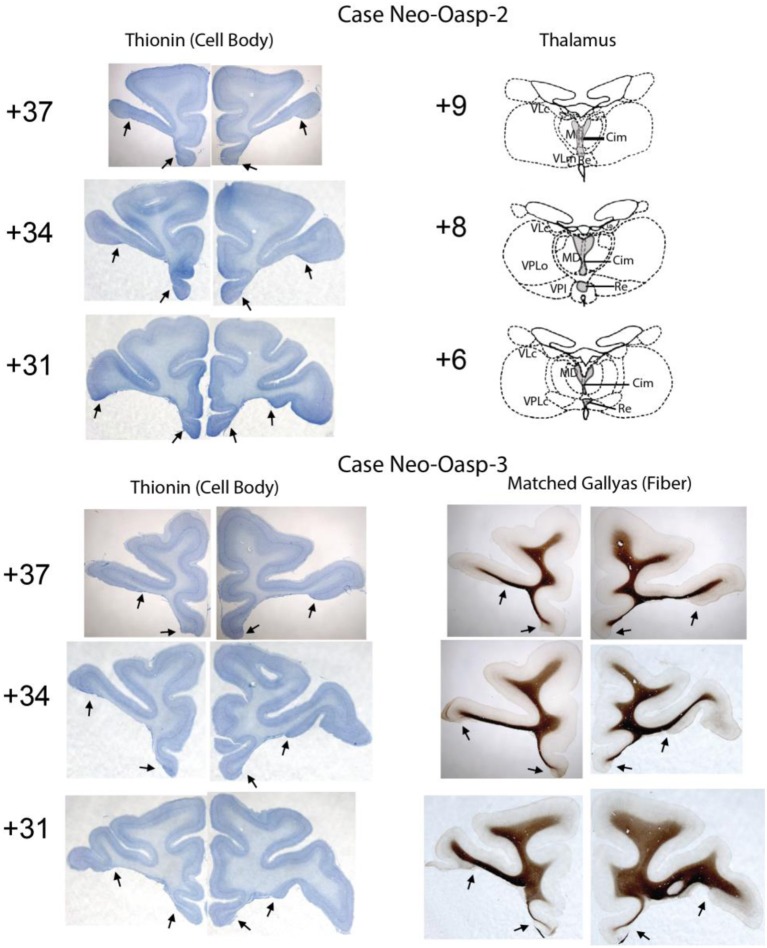
**Extent of neonatal OFC lesions is illustrated on thionin-stained sections for Cases Neo-Oasp-2 and -3 on the left column**. Resulting thalamic degeneration following the neonatal OFC lesions is illustrated on drawings at three levels through the thalamus for a Case Neo-Oasp-2 and sparing of fibers underlying the OFC lesions is illustrated on Gallyas-stained sections for Case Neo-Oasp-3. Abbreviations: Cim, central intermedial; MD, mediodorsal; Re, reuniens; VLc, ventral lateral caudal part; VLm, ventral lateral, medial part; VPL, ventral posterior, lateral part; VPLo, ventral posterior, lateral oral part.

At completion of behavioral testing, at the age of 8–10 years, all animals with neonatal orbital lesions were given a lethal dose of sodium pentobarbital and perfused intracardially with 0.9% saline followed by 4% paraformaldehyde. The brain was removed, post-fixed in 30% sucrose-formalin, and then cut frozen at 50 μm in the coronal plane. Every 10th section was mounted for staining with thionin, providing one section every 0.5 mm, and every 20th section was mounted for staining with silver (Gallyas, 1979), providing one section every 1 mm. The two series of sections were mounted, de-lipidated in Xylene, stained with thionin or gallyas for visualization of cell bodies and fibers, respectively, and cover slipped. For each animal, all sections through the extent of the orbital frontal lesion were microscopically examined and digitized. Estimates of the extent of lesion were plotted at 1-mm intervals through the extent of the entire lesion for each case onto standardized, coronal drawings of the normal macaque brain. Thionin-stained photomicrographs at three levels through the extent of the orbital frontal lesions are illustrated on Figures 1, 2 for all five cases. Representative sparing of orbital frontal white matter is illustrated on the Gallyas-stained sections of case Neo-Oasp-3 and retrograde thalamic degeneration in the thalamus is plotted on drawing of coronal sections of the normal macaque brain for case Neo-Oasp-2 (See Figure 2).

For all cases damage to orbital frontal areas 11 and 13 was extensive and symmetrical, as we had already demonstrated in previous reports using *in vivo* neuroimaging investigation of the lesions (see Table 1). Unintentional damage to adjacent cortical areas was moderate and bilateral for insular area Ia, and minor and mostly unilateral for areas 14 and 12 (See Figures 1, 2). Retrograde thalamic degeneration was found in all cases, with moderate bilateral cell loss in the dorsomedial portion of the magnocellular division of the medial dorsal nucleus and a small patch of dense cell loss in the ventromedial portion of the anterior medial nucleus. Partial cell loss could also be detected in all cases in the central intermedial nuclei as well as in the medial portion of the reuniens nucleus. The distribution of the retrograde degeneration in Group Neo-Oasp thus corresponds to the nuclei that are known to be the main sources of thalamic inputs to orbital frontal cortex (Goldman-Rakic and Porrino, 1985; Barbas et al., 1991; Morecraft et al., 1992; Ray and Price, 1993).

## Experiment 1: reinforcer devaluation paradigm

Damage to orbital frontal areas 11 and 13 in adult monkeys results in severe impairment in flexible decision making as assessed with the reinforcer devaluation task (Machado and Bachevalier, 2007a) while sparing performance on object reversal task (Kazama and Bachevalier, 2009). Yet, very little is known on the long-term effects of orbital frontal damage occurring in infancy when the prefrontal cortex is not yet fully mature. In an earlier report, we demonstrated that, like the adult-onset lesions, neonatal-onset lesions of orbital frontal areas 11–13 did not alter performance on the object reversal task (Kazama and Bachevalier, 2009). To assess whether or not the same neonatal orbital frontal lesions will alter flexible decision making as adult-onset lesions do, Experiment 1 assessed performance of the same experimental and control animals in the reinforcer devaluation task. Monkeys began testing at 3–4 years of age and were re-tested at 5–6 years using methods developed to examine performance of monkeys that had received similar operations in adulthood (Malkova et al., 1997; Machado and Bachevalier, 2007b) and identical to those described in a recent developmental study examining the effects of early damage to the amygdala (Kazama and Bachevalier, 2013).

### Reinforcer devaluation task

#### Apparatus and stimuli

Animals were tested in a Wisconsin General Testing Apparatus (WGTA), fitted with a tray containing three food wells. The two lateral wells in which rewards could be hidden were utilized during testing. One hundred-twenty objects used in prior studies (Machado and Bachevalier, 2007a) were paired to form 60 pairs of easily discriminable objects matched for size. Within each pair of objects, one (S+1 or S+2) was placed over the lateral well of the tray baited with either a peanut, a raisin, or a banana flavored pellet (based on individual preferences indicated by prior behavioral testing). The unrewarded object of the pairs (S-) was located above the other lateral and empty food well. The same pairs of objects were used when animals were re-tested at the later age.

#### Phase I—concurrent discrimination learning

The 60 object pairs were presented sequentially at 30-s intervals for 60 trials per day, 30 with S+1 objects and 30 with S+2 objects, intermixed. Animals were tested daily until the animal reached criterion (90 correct responses in 5 consecutive days). The total number of daily sessions to criterion measured discrimination learning and the number of S+1 or S+2 stimuli selected during the first day of training provided a mean to assess any initial bias toward one type of baited objects. Finally, similar to previous studies, the amount of errors committed prior to criterion was used as a primary measure of performance.

#### Phase II—reinforcer devaluation

Upon reaching criterion during the acquisition phase, animals were then presented with four probe test sessions. During these probe tests, only the rewarded objects of Phase I (S+1 and S+2) were paired (e.g., S+peanut against S+raisin), forming 30 trials per test session. The S+ pairs did not vary across the four sessions, although their left/right positions were altered according to a pseudo random schedule. There were two Baseline test sessions during which the 30 S+ pairs were presented sequentially with 30-s inter-trial intervals. There were also two Devaluation sessions during which just prior to testing, each animal received 100 g of either Food 1 (their 1st preferred food reward) or Food 2 (their 2nd preferred food reward) in the home cage and was allowed to eat freely for 30 min. If the 100 g were consumed, additional food was provided every 15 min until 5 min elapsed without further ingestion of the food reward. Immediately following selective satiation, the animal was transported to the WGTA and tested similar to Baseline sessions (30 pairs of S+ objects). The sequence of presentation of these four test sessions was Baseline I, Devaluation I (Food 1), Baseline II, and Devaluation II (Food 2).

One regular 60-trial Stage I training session intervened between each of the four sessions to ensure that the effects of a reinforcer devaluation condition did not carry over from 1 day to the other, and 2 days of rest followed each of the reinforcer devaluation sessions.

The effects of the lesions on the Devaluation Sessions were assessed using several measures consistent with previous studies (Malkova et al., 1997; Izquierdo et al., 2004; Machado and Bachevalier, 2007a,b): (1) animal's weight (kg) before each devaluation probe session, (2) total food consumed (g) during selective satiation, and (3) time (min) taken to reach satiation. Object/food preferences were determined using the baseline scores. For each Devaluation session, the number of S+1 and S+2 objects selected were recorded as well as whether or not each rewarded food item was ingested by the animal. For both the selection of the objects associated with the satiated food reward, as well as the consumption of the satiated food reward, difference scores were calculated by subtracting the sum of the two baseline scores from the sum of the two satiation scores. The object difference scores indicated the degree to which each subject altered their preferred choice of objects, based on satiation (i.e., select the object associated with the non-satiated food). The food difference scores indicated to what degree each subject continued to consume the devalued food after the object was displaced.

### Statistical analyses

#### Phase I

For the concurrent discrimination learning phase at 4 years, one sample *t*-tests evaluated whether all animals started at chance levels (30/60 correct) and independent *t*-tests were used to analyze group differences for total trials and errors to criterion. Additional repeated measures ANOVA was used to examine group differences in learning objects associated with each reward contingencies (Group × Reward Contingencies). When re-tested at 6 years of age, re-acquisition of the 60 discrimination pairs was analyzed with a repeated measure ANOVA (Group × Age) for total trials and errors.

#### Phase II

Performance of the baseline tests was analyzed for both ages separately using paired samples *t*-tests to assess whether or not animals demonstrated a significant preference for items associated with a specific food reward (S_#_1 or S_#_2). Repeated measures ANOVAs (Group × Age) were conducted on all satiation variables as well as on both object difference scores and food difference scores to assess any changes in performance with age.

In addition, to assess any sparing of functions following the neonatal lesions as compared to adult-onset lesions, scores obtained at 4 years of age were compared to those of adult animals that had received similar aspiration lesions of areas 11 and 13 in adulthood and were tested in the same way at 4 years of age (Machado and Bachevalier, 2007a), using Two-Way ANOVAs (Group × Time at lesions).

Finally, to test whether the effects of neonatal OFC lesions were similar to those of neonatal amygdala lesions on learning the 60 discrimination problems and on flexible choice selection, we compared the errors to criterion to learn as well as the difference scores during devaluation sessions obtained in Groups Neo-C and Neo-Oasp to those reported in animals that had received neonatal amygdala lesions (Group Neo-Aibo) and were tested in the same way (Kazama and Bachevalier, 2013). One-Way ANOVA were used for these comparisons.

For all Two-Way ANOVAs with repeated measures, degrees of freedom for within subjects factors were corrected with the Huynh-Feldt Epsilon when appropriate as indicated in the text.

Effect sizes are provided in all cases where the data revealed either significant or trend-like differences. Finally, given the small number of males and females in each neonatal group and the lack of females in the groups with adult-onset lesions, the factor Sex was not included in any of the statistical tests.

### Results

#### Phase I—concurrent discrimination learning

When tested for the first time at 4 years of age, both groups performed at chance during the initial 60-trials session (*t* = 0.834, *p* > 0.05), indicating no significant initial bias toward the baited objects. All animals reached the learning criterion (90% correct over five sessions) within the limit of testing even though Group Neo-Oasp took longer to learn (1236 trials and 398 errors) than Group Neo-C (720 trials and 232 errors). This group difference did not reach statistical significance for either trials or errors, [*t*_(7)_ = 1.69 and 1.69, *ps* > 0.05, respectively, see Table 2]. Additionally, although rate of learning differed depending on the two types of rewards [Reward contingency effect: *F*_Huynh−Feldt(1, 7)_ = 6.25, *p* < 0.05, μ^2^ = 0.47], the Group effect and the Group × Reward contingency interaction did not reach significance [*F*_(1, 7)_ = 1.27, *p* > 0.05, μ^2^ = 0.15 and *F*_(1, 7)_ = 0.028, *p* > 0.05, μ^2^ = 0.004, respectively], indicating that the relative poorer learning in Group Neo-Oasp was not associated to food-related learning differences. Because the lower performance in the Neo-Oasp group could potentially be related to damage in specific sub-regions of the OFC, a Pearson correlation comparing performance with individual damage to areas 11, 12, 13, and 14 of the OFC was conducted. Results of this analysis did not reveal any statistically significant correlations between performance and damage to individual sub-regions (all *ps* > 0.05).

**Table 2:** **Concurrent discrimination/reinforcer devaluation cognitive scores**.

**Sex**	** Time at test**	**4 years**			**6 years**		
	** Cases**	**Acq**	**Object difference**	**Food difference**	**Retention**	**Object difference**	**Food difference**
	**Neo-C**						
♀	Neo-C-1	226	15	28	53	21	26
♂	Neo-C-2	180	9	18	0	23	24
♀	Neo-C-3	221	16	30	98	19	29
♂	Neo-C-4	301	4	23	93	22	30
	X	232	11	24.8	61	21.3	27.3
	**Neo-Oasp**						
♀	Neo-Oasp-1	479	14	26	119	0	20
♂	Neo-Oasp-2	193	−1	13	22	−5	25
♀	Neo-Oasp-3	588	9	26	253	−4	21.5
♂	Neo-Oasp-4	528	7	23	47	4	19
♀	Neo-Oasp-5	200	8	17	32	4	11
	X	397.6	7.4	21	94.6	−0.2	19.3

*Scores are total number of errors made before criterion days for the acquisition (Acq) of the concurrent discrimination task at 4 years of age and retention of the task 2 years later (6 years). Object difference scores and food difference scores were obtained in the devaluation probe sessions at 4 and 6 years of age. Neo-C, animals with neonatal sham-operations and Neo-Oasp, animals with neonatal OFC area 11/13 lesions. Note that Case Neo-C2 that had been tested in the Devaluation task was not tested on the AX−/BX+ task and was replaced by case Neo-C-5 that had a similar training history*.

When re-tested 2 years later using the exact same stimuli, all animals showed good retention of all stimuli (see Table 2), re-acquiring the task in an average of 270 trials (61 errors) for Group Neo-C and 396 trials (94.6 errors) for Group Neo-Oasp [Age effect, *F*_(1, 7)_ = 39.29, 46.37, *p* < 0.001, μ^2^ = 0.85, 0.87, for trials and errors, respectively]. There were no effect of group [*F*_(1, 7)_ = 1.51, 2.003, *ps* > 0.05, for trials and errors, respectively] and no significant interactions (all *ps* > 0.05).

#### Phase II: reinforcer devaluation

***General satiation variables***. Both groups took similar amounts of time to reach satiation criterion with Food #1 [Group: *F*_(1, 7)_ = 0.003, *p* > 0.05, Age: *F*_(1, 7)_ = 0.072, *p* > 0.05, and no interaction *p* > 0.05] and with Food #2 [Group: *F*_(1, 7)_ = 1.25, *p* > 0.05, Age: *F*_(1, 7)_ = 1.63, *p* > 0.05, and no interaction, *p* > 0.05]. Similarly, for amount of Food # 1 and Food # 2 consumed during the satiation, there were no significant effects of Group [*F*_(1, 7)_ = 0.06 and 3.11, *ps* > 0.05]. However, although the Age effect did not reach significance for Food # 1 [Age effect: *F*_(1, 7)_ = 1.16, *p* > 0.05], it did for Food # 2 [*F*_(1, 7)_ = 5.42, *p* = 0.05, μ^2^ = 0.44]. In addition, although there were no significant interactions for Food # 1 (all *ps* > 0.05), the Age × Group interaction was significant for Food # 2 [*F*_(1, 7)_ = 17.13, *p* = 0.004, μ^2^ = 0.71], indicating that Group Neo-C consumed greater amounts of Food # 2 relative to Group Neo-Oasp at the later age point (*t* = 2.65, *p* = 0.03).

Finally, as expected, all animals gained approximately a kilogram of body weight [Age: *F*_(1, 7)_ = 21.51, *p* = 0.006], however the Group effect was not significant [*F*_(1, 7)_ = 1.48, *p* > 0.05] with no significant interaction (*p* > 0.05).

***Baseline probe sessions***. Paired samples *t*-tests comparing selection of S^+^#1 vs. S^+^#2 objects for each group at both ages revealed that all animals had a significant preference for objects associated with a specific reward during baseline trials (e.g., selection of more peanut items than raisin items) [Age 4: *t* = 4.131 and 2.726, *ps* = 0.05, Age 6: *t* = 5.29 and 4.71, *ps* < 0.05, for Groups Neo-C and Neo-Oasp, respectively). Thus, the effects of Group and Age did not reach significance [*F*_(1, 7)_ = 3.30, 1.46, *ps* > 0.05, respectively], nor did any of the interactions (all *ps* > 0.05).

**Figure 3:**
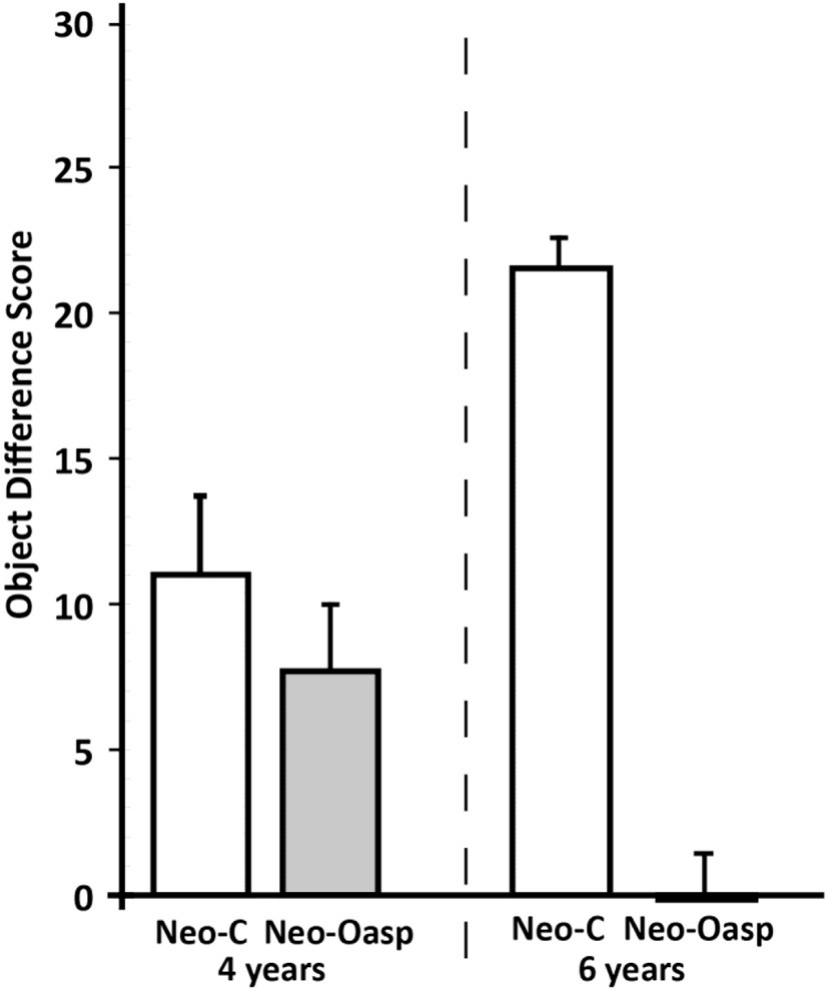
**Object difference scores in animals with neonatal OFC lesions (Neo-Oasp) and sham-operated controls (Neo-C) at the two ages tested**. Object difference score measures how often an animal chooses an object paired with a devalued food item during the satiation probe session as compared to baseline session. Vertical bars provide s.e.m values.

***Reinforcer devaluation probe sessions***. The satiation object difference scores for each animal (Table 2 and Figure 3) were calculated by subtracting the number of objects associated with each food in the baseline sessions and the number objects associated with that same food in the devaluation session when that food had been devalued. Thus, a high object difference score indicates that the animal selected more satiated food-related objects during baseline than during the devaluation session, and therefore demonstrated greater flexibility. As shown in Figure 3, animals with Neo-Oasp lesions demonstrated less flexibility than controls as revealed by significant lower object difference scores at both ages [Group: *F*_(1, 7)_ = 30.17, *p* = 0.001, μ^2^ = 0.81]. In addition, the significant Group × Age interaction [*F*_(1, 7)_ = 18.92, *p* = 0.003] indicated that while Group Neo-C showed greater flexibility at 6 years than at 4 years [*t*_(6)_ = −3.50, *p* < 0.02], Group Neo-Oasp showed the reverse, i.e., less flexibility at 6 years than at 4 years [*t*_(8)_ = 2.47, *p* < 0.04].

The satiation food selection difference scores (see Table 2) measured the degree to which the animal actually took and ingested the devalued food after displacing the object. Thus, animals with large food difference scores indicated a refusal to eat the satiated food after the object was displaced. As compared to Group Neo-C, Group Neo-Oasp consumed greater amounts of satiated food, [*F*_(1, 7)_ = 5.35, *p* = 0.054], although there was no effect of Age [*F*_(1, 7)_ = 0.033, *p* > 0.05], and no significant interaction [all *p* > 0.05]. The data suggest that, after displacing objects associated with satiated foods, animals with early damage to the OFC had a greater tendency to ingest the satiated food reward.

### Comparisons between early-onset vs. late-onset OFC lesions

For these analyses, scores obtained for animals with neonatal lesions obtained when they were tested for the first time at 4 years of age were compared to those of a previously published study examining animals with similar adult-onset lesions (adult sham-operated controls and adult OFC-operated animals, *n* = 3 in each group) also tested for the first time at 4 years of age (Machado and Bachevalier, 2007a).

#### Phase I—acquisition

**Figure 4:**
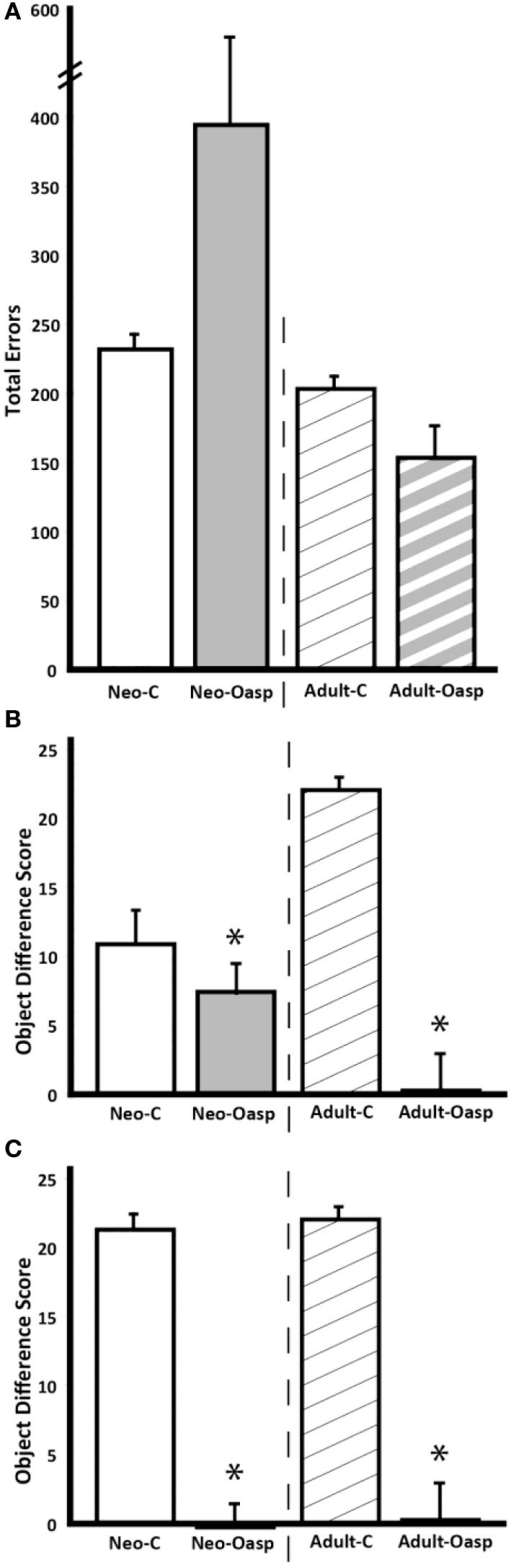
**(A)** Mean number of errors (± s.e.m) made before reaching criterion in the concurrent discrimination task for animals with neonatal lesions (Neo-C and Neo-Oasp) and for animals with adult-onset lesions [Adult-C and Adult-Oasp; data are from Machado and Bachevalier (2007b)] that had learned the task for the first time at the age of approximately 4 years. Criterion was set at 90% correct or better over 5 consecutive days. **(B,C)** Averaged object difference scores (± s.e.m) for animals with neonatal lesions (Neo-C and Neo-Oasp) and for animals with adult-onset lesions (Adult-C and Adult-Oasp). In **(B)**, scores of animals with neonatal lesions tested for the first time at 4 years and in **(C)**, scores of animals with neonatal lesions tested for the second time at 6 years. ^*^p < 0.05.

All animals learned the 60 discrimination problems at the same rate regardless of timing of lesion [Group: *F*_(1, 11)_ = 0.733, *p* > 0.05; Time at lesions: *F*_(1, 11)_ = 3.15, *p* > 0.05; Group × Time at lesions: *F*_(1, 11)_ = 3.15, *p* > 0.05; see Figure 4C]. Although the group effect did not reach significance for errors to criterion [Group Effect: *F*_(1, 11)_ = 0.971, *p* > 0.05], Time at lesion effect did reach significance [*F*_(1, 11)_ = 4.78, *p* = 0.05, μ^2^ = 0.30], but the Group × Time at lesion interaction did not [*F*_(1, 11)_ = 2.88, *p* > 0.05]. This indicates that overall animals with neonatal lesions made more errors than those with adult-onset lesions [*t*_(13)_ = 2.13, *p* = 0.053], although this difference was mostly driven by an increased number of errors in three of the five animals in Group Neo-Oasp (see Figure 4).

#### Phase II—devaluation

Comparisons of the object difference scores that animals of the neonatal-onset lesion groups obtained at 4 years with those of the animals of the adult-onset lesion group (Figure 4B) revealed that damage to areas 11 and 13 resulted in significantly lower Object Difference scores, hence less flexible decision-making for Groups Oasp [Group: *F*_(1, 11)_ = 24.53, *p* < 0.001, μ^2^ = 0.69], as compared to controls. Although Timing at lesions did not reach significance [Time at lesion effect: *F*_(1, 11)_ = 0.485, *p* > 0.05], the interaction Group × Time at lesion did [*F*_(1, 11)_ = 12.67, *p* < 0.005], indicating that Group Neo-C showed less flexibility than Group Adult-C [*t* = −3.85, *p* < 0.03], whereas Group Neo-Oasp did not differ from Group Adult-Oasp [*t* = 1.92, *p* > 0.05]. Given that flexible choice selection improved significantly in Group Neo-C from the first time they were tested at 4 years to the second time at 6 years, we also compared Object Difference scores when the animals with the neonatal lesions were tested at 6 years with those of the animals with adult lesions (see Figure 4C). At this later age, animals in both Groups Neo-C and Neo-Oasp performed similarly to those in the adult groups as revealed by a significant group effect [*F*_(1, 11)_ = 139.25, *p* < 0.001] but no significant interaction [*F*_(1, 11)_ = 0.02, *p* > 0.05].

### Comparisons between neonatal OFC lesions and neonatal amygdala lesions

As reported above, animals with Neo-Oasp lesions were slightly retarded in learning the 60 problems (average: 398 errors) but those with Neo-Aibo (average: 199 errors) learned as rapidly as controls (average: 232 errors). This group difference reached significance [*F*_(2, 12)_ = 4.00, *p* < 0.05] and *post-hoc* analyses indicated that Group Neo-Aibo learned as rapidly as Group Neo-C (*p* > 0.05), but only Group Neo-Aibo learned faster than Group Neo-Oasp (*p* < 0.02).

Furthermore, there was a significant group effect for difference scores obtained in the devaluation task [*F*_(2, 12)_ = 9.85, *p* < 0.003]. *Post-hoc* analyses indicated that both Groups Neo-Oasp and Neo-Aibo obtained similar scores (*p* > 0.05) but both groups obtained devaluation scores significantly lower than those of Group Neo-C (all *ps* < 0.02).

### Summary of results for Experiment 1

The data indicate that neonatal damage to areas 11 and 13 resulted in a slight retardation in initially learning the large 60 S+/S− set of stimuli with three of the five animals making twice more errors than all four controls. However, this group difference did not reach statistical significance and the individual difference between animals of Group Neo-Oasp did not seem to correlate with extent of damage to areas 11 and 13 or even with inadvertent damage to adjacent OFC fields. In addition, the satiation object difference scores increased significantly in control animals from 4 to 6 years, reflecting most likely stronger flexible choice selection with repeated training. By contrast, the satiation object difference scores for animals with neonatal OFC lesion worsened with age. Finally, there were two additional findings of note that demonstrated similar effects of the early-onset and late-onset OFC lesions (Machado and Bachevalier, 2007a). First, both early- and late-onset OFC lesions resulted in an inability to flexibly shift choices away from objects associated with devalued foods, although the similar effects of timing of the OFC lesions was stronger when animals with early-onset lesions were tested for the second time at 6 years. Second, both early- and late-onset OFC lesions increased animals' tendency to ingest the satiated food rewards once the objects had been displaced. Taken together, the data suggest that areas 11 and 13 are required for the development of flexible decision-making and no other brain structures could compensate for the deficits in flexible decision-making after neonatal damage to OFC areas 11 and 13. In addition, the results also strengthened those already reported with adult-onset lesions (Baxter et al., 2000). To assess whether this lack of behavioral flexibility after neonatal OFC lesions observed with appetitive task will also be present under aversive conditions, in Experiment 2 we examined performance of these same animals on the AX+/BX− fear-potentiated startle paradigm.

## Experiment 2: AX+/BX− fear-potentiated startle paradigm

Given that results of Experiment 1 indicated that neonatal damage to OFC areas 11 and 13 resulted in significant impairment in flexible changes in food choice, we then tested whether these same neonatal-onset OFC lesions would also alter the ability to flexibly modify fear reactivity when cues signal safety. Although there exist no data on the effects of adult-onset OFC lesions on fear conditioning, condition inhibition, and extinction in monkeys, reports in rodents and humans (Gewirtz et al., 1997; Schiller et al., 2008) have provided mixed results regarding the evidence for a contribution of the ventral prefrontal cortex in condition inhibition and extinction. Thus, at completion of second round of testing on the Reinforcer Devaluation task, animals of Experiment 1 were tested in the AX+/BX− paradigm to assess their abilities to condition to fear and safety cues, to use safety cue to modify that fear reactivity to the fear cue (condition inhibition) and to extinguish their fear reactivity when the fear cue was not paired with the aversive stimulus. Note that all five animals with Neo-Oasp lesions but only three of the four animals in Group Neo-C participate in this experiment. Thus, case Neo-C-2 that had participated in Experiment 1 was replaced in Experiment 2 by case Neo-C-5 that had the same behavioral training history to the remaining animals in both Groups Neo-C and Neo-Oasp.

### AX+/BX− paradigm

Training began when the animals were 6-7 years of age and lasted approximately 1 month. All inter-session intervals were 72 h, and session length depended upon the stage of training (see below for details). Animals were given their normal daily chow, water, and fresh fruit, as well as additional treats during primate chair training. All methods have been detailed in earlier reports (Winslow et al., 2002, 2008; Antoniadis et al., 2007; Kazama et al., 2012), and will be briefly described below.

#### Apparatus

Animals were seated in a non-human primate chair located in a sound attenuated chamber equipped with an automated system designed to deliver unconditioned and conditioned stimuli. The chair was positioned above a load cell (Med Associates, St. Albans, VT). Movements initiated by the animals produced displacement of the load cell (Sentran YG6-B-50KG-000), the output of which was amplified, and analyzed via the Med Associates Primate Startle Software (Med Associates, St. Albans, VT).

#### Stimuli

Two unconditioned stimuli (US) were used. A 500 ms jet of compressed air (100 PSI) generated by an air compressor located outside the chamber and projected at the face of the monkey via four air jet nozzles. A startle stimulus, which was a 50 ms burst of white noise of varying intensities (range: 95–120 dB) delivered through the same speakers as the back ground noise. Three cues served as either an aversive conditioned stimulus (A), a safety conditioned stimulus (B) or a neutral stimulus (X). The visual CS was a 4 s light produced by 4 overhead halogen bulbs producing a combined 250 Lux, attached to the top of the test chamber. The auditory CS was an 80 dB, 4 s, 5000 kHz tone produced by an overhead speaker. The tactile CS was produced by a quiet computer fan that directed gentle airflow onto the monkey's head. The CS assignments as cues A, B or X were pseudo-random and counter-balanced across groups. Thus, some animals received the light as the aversive CS, whereas others received the tone as aversive CS, and so forth.

#### Acoustic startle response

To evaluate any potential effects of lesion on acoustic startle, the animals were placed in the apparatus and exposed on 2 separate days of 60 trials each, which were composed of baseline activity without startle stimuli (10 trials), and of startle responses to startle eliciting noise bursts of varying intensities (95, 100, 110, 115, and 120 dB; 10 trials each). All trials were pseudo-randomly intermixed throughout each session. Animals were then tested for pre-pulse inhibition before moving on to the AX+/BX− paradigm (Heuer et al., 2010). Data for pre-pulse inhibition will be reported separately.

#### Pre-training

Prior to the conditioning phase, the animals were habituated to the three conditioned cues to assess any unconditioned effects of the cues on the startle response prior to conditioning. First, animals received 2 separate days of 30 trials each during which the to-be-conditioned cues (light, tone, or airflow from quiet fan) and their combinations (light/tone, light/airflow, tone/airflow) were presented in the absence of the startle noise. Then, animals were given days of 60 trials, consisting of 30 trials with the startle noise alone (95 dB), and 30 trials in which the 95 dB startle noise was elicited in the presence of one of the to-be-conditioned cues or their combinations for 5 trials each pseudo-randomly ordered. Within each of the cue-startle trial the startle stimulus was presented 4 s after the onset of the CS. These pre-training sessions were repeated for each monkey until presentation of the cue that was assigned to serve as the safety signal (cue B) for that animal produced less than a 30% increase in startle amplitude compared to startle stimulus alone (noise alone) presentations.

#### A+ training phase

The purpose of this phase was to train the animal, using Pavlovian fear conditioning procedures, to associate a cue (A+) with an aversive air-blast. These A+ air-blast trials occurred four times per 28-trial session, and were always scheduled such that one occurred at the beginning and one at the end of each session. The remaining two pairings were pseudorandomly intermixed within the 24 startle test trials across sessions so that animals could not predict when cue A would be followed by an air-blast as opposed to a startle stimulus. The startle stimulus or air-blast was presented 4 s after the onset of cue A. The remaining 24 trials consisted of four trial-types (Noise Alone 95 dB, Noise Alone 120 dB, Cue A 95 dB Noise, Cue A120 dB Noise) and were presented pseudo-randomly six trials each per session. Animals received A+ Training for a minimum of two sessions, and until their percent Fear-Potentiated Startle (% fear-potentiated startle) was 100% above their pre-training startle in the presence of the A cue. Percent fear-potentiated startle was defined as: [Mean startle amplitude on CS test trials – mean startle amplitude on startle noise alone test trials)/mean startle amplitude on noise burst alone test trials] × 100.

#### A+/B− training phase

The purpose of this phase was to train the animal to associate a second cue (B) with the absence of an air-blast (B−), thus this cue was termed the safety-signal. Animals received 40-trial sessions composed of six trials in which both startle noise intensities (95 dB and 120 dB) were given in the presence of the safety cue B, which was never paired with the air-blast US; four trials in which cue A continued to be paired with the air-blast (according to the schedule described previously—A+) or both startle noise intensities (95 dB and 120 dB, six trials each) given in the presence of cue A or alone (six trials each). Animals received A+/B− Training for a minimum of two sessions, and until a difference of 100% fear-potentiated startle was obtained between the two cues.

#### AX+/BX− training phase

**Figure 5:**
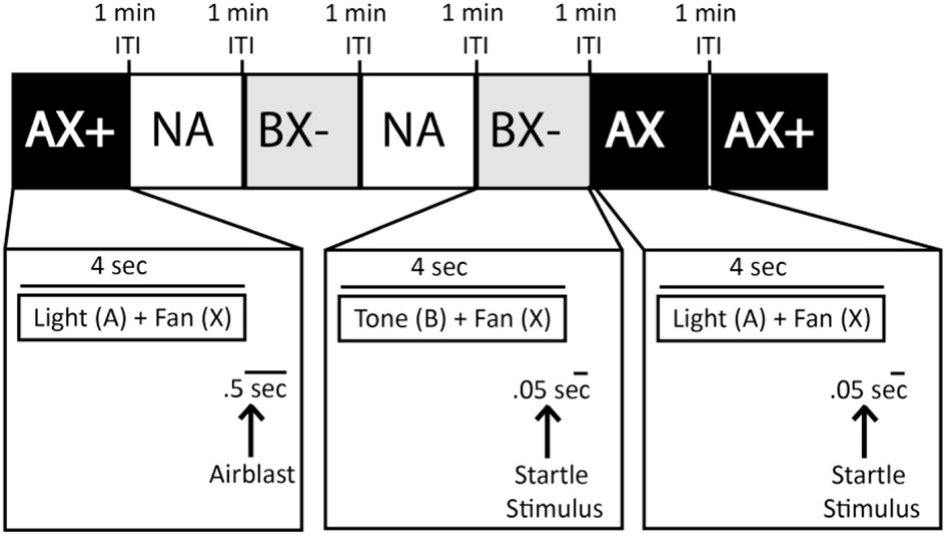
**Sample of AX+/BX− training session testing block**. Squares represent various trial types (e.g., AX+, AX, Noise Alone (NA), BX−) within a training session. Trials were separated by a 1 min inter-trial intervals (ITI).

Previous conditioned inhibition training in humans using the typical design (A+/AB−) indicated that B, the safety signal, did not transfer to another cue that had not previously been put in compound with A and instead AB− was probably not treated as a compound cue consisting of the aversive and safety cues, but rather as a completely novel third cue (Grillon and Ameli, 2001). Thus, the purpose of this phase was to train the animal to discriminate compound cues using a third neutral cue (X), which was presented in combination with both the A+ or B− cues. This phase included 40-trial sessions constructed similarly to A+/B− Training. The only difference is that both the aversive cue (A+) and the safety cue (B−) were presented in combination with the neutral cue (X), yielding compound cues AX+ and BX− (see Figure 5). As with the A+/B− Training, animals received the AX+/BX− Training for a minimum of two sessions, and until there was a difference of 100% fear-potentiated startle between the two compound cues.

#### AB testing/transfer test

Animals were tested for conditioned inhibition (i.e. transfer) in a single session within 72 h after the last AX+/BX− training session to examine the potential inhibitory effects of B on A. This 48-trial probe session consisted of all trial types, including two A+ air-blast pairings intermixed within (a) 95 dB and 120 dB Noise Alone trials (6 trials each), (b) 95 dB and 120 dB startle stimuli in the presence of each of the various cue and cue compounds (A, B, AX, BX, 5 trials each per noise intensity), and (c) in the presence of the novel AB cue (5 trials per noise intensity). Hence, when trained in this way transfer of fear on the AB test trial could not be accounted for by configural learning. All trials were pseudo-randomly intermixed.

#### Extinction

Finally, all animals were presented with successive 12-trial sessions of the 95 dB startle stimulus elicited alone (4 trials) or in the presence of cues A and AX to evaluate fear extinction (4 trials of each type). Training was completed when the animal returned to its pre-training startle amplitude.

### Data analysis

Throughout the different phases, the startle amplitudes were recorded. Data analysis included three parts. First, we used a Huynh-Feldt corrected repeated measures ANOVA to compare the acoustic startle responses to the varying intensities (95, 100, 110, 115, and 120 dB) across groups. Second, we assessed the animal's ability to associate and discriminate between the aversive and safety cues (A, B, AX, BX) using a “sessions to criterion” measure. Because all our control animals learned the task at floor (e.g., two sessions per phase), and thus had no variability, the group differences were analyzed with non-parametric statistics (Mann–Whitney U). Third, because previous reports (Winslow et al., 2008) indicated that startle values are not normally distributed; we transformed the transfer test data using a logarithmic base 10 transformation and compared both groups using a Huynh-Feldt repeated measures ANOVAs. Finally, to assess whether the effects of neonatal OFC lesions differ from those obtained earlier after neonatal amygdala lesions, we compared the fear conditioning scores to cue A (see Table 4) and modulation of fear probe trial (see Table 5) of Group Neo-Oasp to those reported earlier after neonatal amygdala lesions (Kazama et al., 2012), using One-Way ANOVA and Two-Way ANOVA with repeated measures, respectively.

### Results

#### Acoustic startle response

**Figure 6:**
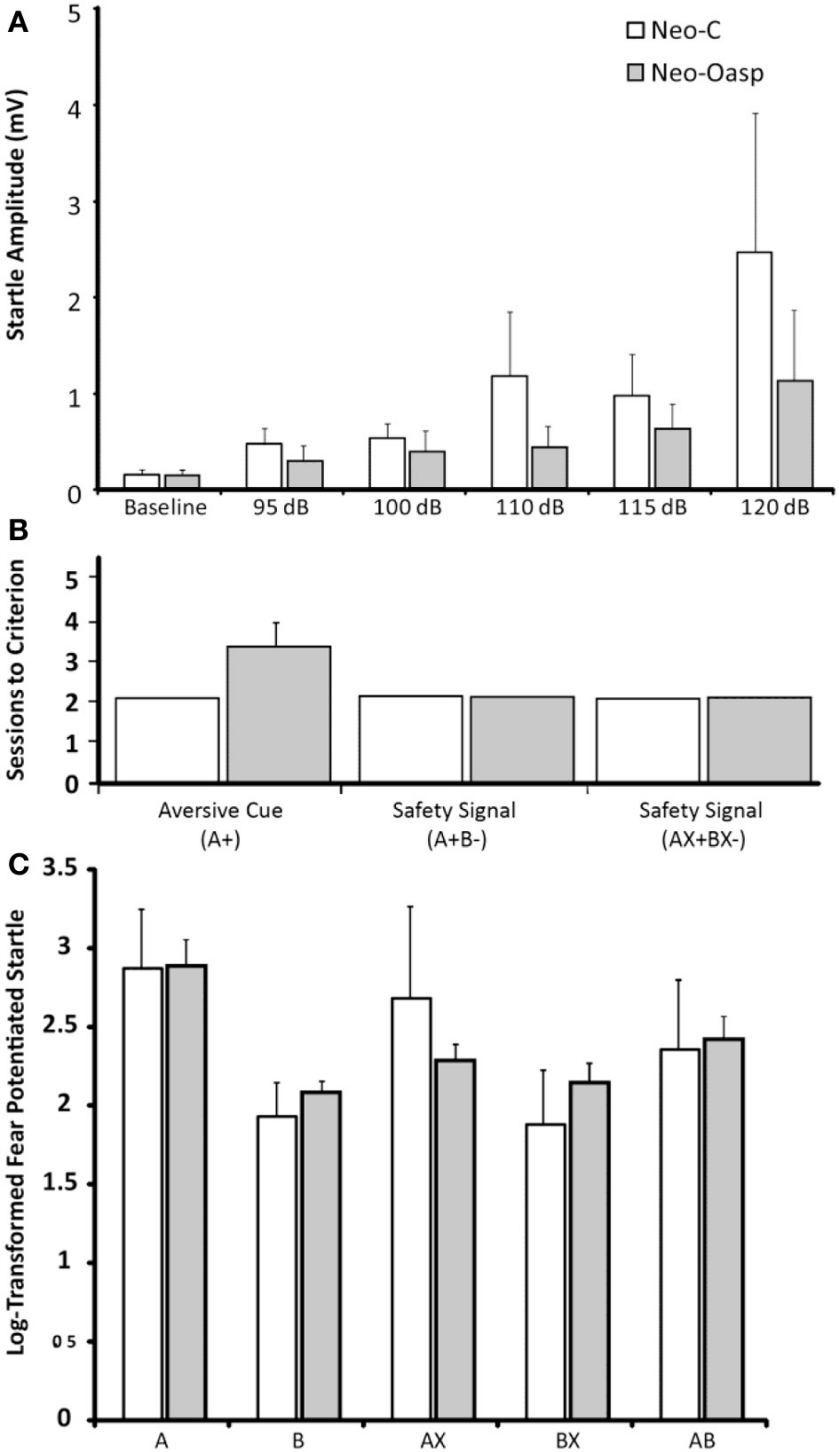
**(A)** Mean Acoustic Startle Response to differing sound intensities (95 dB, 100 dB, 110 dB, 115 dB, and 120 dB) for Group Neo-C (White bars) and Group Neo-Oasp (Gray bars). **(B)** Average sessions to criterion per stage of learning for Group Neo-C and Group Neo-Oasp. **(C)** Average log-transformed % fear-potentiated startle by cue for Neo-C and Group Neo-Oasp. Although there was no significant effect of group, all aversive cue types were significantly different from all safety cues, and both cue types were significantly different from the transfer cue (AB), with the exception of Group Neo-Oasp, cue AX, which was not significantly different from the safety cues (*p* > 0.05, all other *ps* < 0.05). Error bars represent the s.e.m for each group.

Because the baseline startle response of two animals in the control group (cases Neo-C-2 and Neo-C-6) was greater than the maximum amplitude of the load cell, these two animals were dropped from the study. As illustrated in Table 3 and Figure 6A, both sham-operated and animals with neonatal OFC lesions demonstrated greater startle responses with increasing startle noise intensity [Startle amplitude effect: *F*_Huynh−Feldt(1, 4)_ = 6.75, *p* = 0.01). In addition, although the Group effect and the Group × Startle amplitude interactions did not reach significance [*F* = 2.37 and *F* = 1.42, all *ps* > 0.05, respectively], startle amplitudes across almost all noise intensities were slightly lower in animals with Neo-Oasp lesions than in sham-operated controls. Obviously, this effect would have been even more pronounced if the two control animals, at the ceiling of the measurement scale at all intensities, had been included.

**Table 3:** **Raw baseline acoustic startle curve**.

**Sex**	**Group**	**Baseline**	**95 dB**	**100 dB**	**110 dB**	**115 dB**	**120 dB**
♀	Neo-C-1	0.14	0.76	0.59	0.83	1.16	4.40
♀	Neo-C-3	0.15	0.39	0.60	2.59	1.75	4.04
♂	Neo-C-4	0.10	0.22	0.23	0.52	0.41	0.37
♀	Neo-C-5	0.26	0.55	0.73	0.78	0.61	1.07
	X	0.16	0.48	0.54	1.18	0.98	2.47
♀	Neo-Oasp-1	0.14	0.21	0.20	0.19	0.55	0.65
♂	Neo-Oasp-2	0.11	0.22	0.61	0.47	0.92	2.56
♀	Neo-Oasp-3	0.15	0.16	0.16	0.17	0.15	0.26
♂	Neo-Oasp-4	0.13	0.21	0.19	0.26	0.23	0.42
♀	Neo-Oasp-5	0.27	0.81	0.83	0.93	0.95	1.66
	X	0.16	0.32	0.40	0.40	0.56	1.11

*Scores are mean raw startle amplitudes taken during the initial baseline acoustic startle sessions*.

#### Fear learning (A+ training)

All animals, regardless of lesion groups learned to associate Cue A+ with the air-blast very quickly. Control animals all performed at floor, completing this stage in the minimum two sessions, whereas animals in Group Neo-Oasp took an average of 3.4 sessions; a group difference that did not reach statistical significance (Mann–Whitney *U* = 6.50, *p* > 0.05, Table 4, Figure 6B).

**Table 4:** **Sessions per learning stage**.

**Sex**	**Group**	**A+**	**A+B−**	**AX+BX−**	**Combined safety learning**	**Extinction**
♀	Neo-C-1	2	2	2	4	5
♀	Neo-C-3	2	2	2	4	5
♂	Neo-C-4	2	2	2	4	2
♀	Neo-C-5	2	2	2	4	2
	X	2	2	2	4	3.5
♀	Neo-Oasp-1	2	2	2	4	3
♂	Neo-Oasp-2	2	2	2	4	5
♀	Neo-Oasp-3	5	2	2	4	3
♂	Neo-Oasp-4	5	2	2	4	2
♀	Neo-Oasp-5	3	2	–	–	–
	X	3.4	2	2	4	3.25

*Scores are total number of sessions to reach criterion for the initial fear learning (Stage A+), the safety signal learning stages (A+B−, AX+BX−; Combined Safety Learning is the summed scores of the two safety signal learning stages), and the extinction stage. X, Group means for each stage. Note that Case Neo-Oasp-5 did not complete the task due to behavioral problems*.

#### Fear/safety signal discrimination learning (A+B−, AX+BX− training)

Because both A+B− and AX+BX− phases were theoretically similar in nature, data for these 2 phases were combined for the analyses (see Table 4, Figure 6B). One animal, Neo-Oasp-5 developed very high baseline startles and had to be dropped at the AX+BX− training phase. All remaining animals, regardless of group, learned to differentiate between the aversive and safety cues in the minimum 2 days per stage with no variability between animals (Mann-Whitney *U* = 8.00, *p* > 0.05).

#### Modulation of fear in the presence of the safety signal (AB probe trial)

For the four control animals and four OFC animals that learned to discriminate between the aversive and safety cues, a repeated measures ANOVA was used to assess differences between the log-transformed % fear-potentiated startle to the various cues (i.e., A, B, AX, BX, and AB). As seen in Table 5 and Figure 6C, there were no differences between the two groups [*F*_(1, 8)_ = 0.011, *p* > 0.05], and no interaction between the two factors [*F*_(4, 8)_ = 0.852, *p* > 0.05]. However, both the sham-operated animals (Neo-C) and animals with early OFC damage (Neo-Oasp) had significantly greater startle in the presence of the aversive cue (A) compared to either the safety cues (B, BX; *t*-tests, all *ps* < 0.05) or the aversive cue and the transfer cue (A vs. AB; *t*-tests, all *ps* < 0.05), although animals with early OFC damage did not startle significantly high in the presence of the AX cue relative to BX or AB cues (*t*-tests, all *ps* > 0.05).

**Table 5:** **Log-transformed % fear-potentiated startle**.

**Sex**	**Group**	**A**	**B**	**AX**	**BX**	**AB**
♀	Neo-C-1	3.35	2.07	3.57	2.35	1.9
♀	Neo-C-3	2	1.48	1.77	1.27	1.85
♂	Neo-C-4	3.58	2.46	3.8	2.51	3.54
♀	Neo-C-5	2.57	1.64	1.36	1.23	2.04
	X	2.87	1.91	2.63	1.84	2.33
♀	Neo-Oasp-1	3.33	1.99	1.82	2.53	3.03
♂	Neo-Oasp-2	3.05	2.51	2.47	1.80	2.66
♀	Neo-Oasp-3	2.46	1.86	2.34	2.1	1.63
♂	Neo-Oasp-4	2.71	2.28	2.31	1.97	2.29
♀	Neo-Oasp-5	–	–	–	–	–
	X	2.89	2.16	2.24	2.10	2.40

*Scores are Log-Transformed % fear-potentiated startle amplitudes taken during the transfer test. Each individual score was obtained from the very first time the animal experienced that cue at the optimal decibel level (95 dB or 120 dB) for that particular animal. X, group means for each stage*.

#### Extinction

As seen in Table 4, both groups extinguished very quickly to repeated presentations of the fearful cues (A−, AX−) in the absence of the US, averaging less than four sessions to return to baseline levels of startle (*p* > 0.05).

### Comparisons between the neonatal OFC lesions and neonatal amygdala lesions

The Kruskal Wallis analyses revealed a significant group difference for learning the fear cue [*U*_(2)_ = 6.51, *p* < 0.04], with animals with neonatal amygdala lesions requiring slightly but significantly more trials than animals with OFC lesions or controls (all *ps* < 0.05). In addition, the Two-Way ANOVA comparing groups and scores in all 5 cues in the probe trial revealed no significant group difference [*F*_(2, 9)_ = 0.06, *p* > 0.05] and no Group × Cues interaction [*F*_(8, 36)_ = 0.96, *p* > 0.05]. However, the factor Cue reached significance [*F*_(4, 36)_ = 9.14, *p* < 0.001] indicating that animals in all groups had greater startle for the fear cues (A and AX) than for the combined AB cue (all *ps* < 0.05) and greater startle for the combined AB cue than for the safety cue (B and BX).

### Summary of Experiment 2

The data demonstrated that neonatal lesions of OFC areas 11 and 13 did not alter acquisition of the fear and safety cues, condition inhibition, and extinction. In addition, the intact fear conditioning after neonatal OFC lesions differed from the slight retardation in fear learning reported after neonatal amygdala lesions, although neither lesions affected safety signal learning and the modulation of the fear response in the presence of the safety cue.

## Discussion

The major aim of the study was to characterize the contribution of orbital frontal areas 11 and 13 of the OFC to the development of flexible behavioral modulation, to determine whether early-onset OFC lesions will result in deficits similar to those observed after adult-onset OFC lesions and to assess whether the outcomes of the neonatal orbital frontal lesions paralleled the outcomes reported after neonatal amygdala lesions. The results from the Reinforcer devaluation task revealed that adult animals that had sustained early damage to areas 11 and 13 were only slightly retarded in learning the 60 pairs of discrimination problems and retained these problems over approximately a 2-year period. They also demonstrated normal ability to associate specific stimuli with particular food items. However, they were greatly impaired in flexibly shifting their preferences away from stimuli associated with the devalued food and when displacing the devalued objects, they had the tendency to reach for and ingest the devalued food rewards. These deficits in behavioral flexible became stronger when animals were animals with OFC lesions were tested for a second time at 6 years and at this age their performance was indistinguishable from that of animals that had received the same OFC lesions as adults. In contrast to the impairments observed with the Reinforcer devaluation task, results from the AX+/BX− fear-potentiated startle paradigm indicated that these same Neo-Oasp animals had excellent fear/safety discrimination learning, and more importantly, were generally able to flexibly use safety signals to inhibit their fear response in the presence of safety signals (i.e., A vs. B and AX vs. BX). Most strikingly, each Neo-Oasp animal had lower startle in the presence of cue AB vs. AX, indicating conditioned inhibition to the novel AB compound, comparable to that seen in the Neo-C animals. Additionally, Neo-Oasp animals demonstrated normal behavioral flexibility in their ability to extinguish their startle response in the presence of the AX− stimuli. Taken together, the results suggest that orbital frontal areas 11 and 13 are critical for the development of flexible decision-making, at least under appetitive or rewarding situations, but not for flexibly processing fear and safety signals. These contrasting effects of neonatal orbital frontal lesions will be discussed in turn below and will be compared to results on the effects of early amygdala damage on the same tasks.

### Decision-making behavior after reinforcer devaluation

#### Learning stimulus-reward associations

Although learning scores of animals with Neo-Oasp lesions did not differ statistically from those of sham-operated animals, three of the five animals in Group Neo-Oasp made twice as many errors as the controls did to learn the 60 discrimination problems. This slight retardation in stimulus-reward associations did not correlate positively with the extent of damage to OFC areas 11 and 13 or with inadvertent damage to adjacent OFC fields and contrasts with the normal performance of the same Neo-Oasp animals in simpler version of discrimination tasks using a single pair of objects or even 5 pairs of objects presented concurrently across daily sessions (Kazama and Bachevalier, 2012). The slight retardation in stimulus-reward association learning may be due to either the large number of problems the animals had to learn concurrently in the case of the Reinforcer Devaluation task as compared to 1- or 5-pair discrimination tasks or an inability to maintain the encoding of rewarded objects over long delays, given that as compared to the 1- and 5-pair discrimination tasks, the Reinforcer devaluation task imposed a delay of 24-h between training session. Earlier lesion studies in monkeys have already indicated that orbital frontal cortex lesions in adulthood (Meunier et al., 1997) or in infancy (Pixley et al., 1997; Malkova and Bachevalier, personal communication) impaired recognition of objects when long delays are used between encoding and retrieval. Furthermore, the impairment in learning stimulus-reward associations after early-onset orbital frontal lesions contrasts with the normal performance found after adult-onset lesions (Izquierdo et al., 2004; Izquierdo and Murray, 2007; Machado and Bachevalier, 2007b). The current findings suggest greater impact of the neonatal orbital frontal lesions on discrimination learning. Yet, because 2 animals in Group Neo-Oasp learned as fast as control animals and because all Neo-Oasp animals attained the learning criterion in the limit of training, showed good retention of the 60 problems over a 2-years period, and good memory of the specific food items associated with each positive object, it is possible that the slight learning deficit may be associated to factors others than the lesion itself.

#### Reinforcer devaluation

Neonatal damage to OFC areas 11 and 13 affected the animal's tendency to inhibit selection of objects associated with a devalued reinforcer. This impairment occurred even though the animals were able to associate specific stimuli with specific food rewards, as revealed by their tendency to select objects associated with their preferred food more frequently than objects associated with the other food in the two baseline conditions. The Neo-Oasp lesions slightly increased the tendency of animals to retrieve the rewards after the devalued objects were selected. These impairments became more robust when the animals were tested for the second time and, at that age, strongly paralleled the impairments observed in animals with either permanent or temporary inactivation to OFC areas 11 and 13 performed in adulthood (Machado and Bachevalier, 2007a; Rudebeck and Murray, 2011; West et al., 2012). Thus, the data indicate little, if any, recovery of functions after neonatal orbital frontal cortex lesions.

The impairment in flexibly altering object selection after food devaluation in animals with Neo-Oasp lesions contrasts with their unimpaired performance in object reversal learning (1 pair or 5 pairs, Kazama and Bachevalier, 2012). Although the two tasks measure abilities to modify object selection, there are clear distinctions on the type of information necessary to make the change in selection pattern. In object reversal learning, only one of the two objects is rewarded and animals must inhibit selection of the rewarded object when the reward has been switched without warning to the other object. Thus, animals must extinguish a previously learned response and select a more appropriate one. In the food devaluation test, by contrast, all objects are rewarded but the reward has been devalued for one of the two objects of each pair. The animals must rely on information about changes on their internal state to adjust their response pattern. Thus, impairment in the Reinforcer Devaluation task after neonatal orbital frontal lesions may demonstrate an inability to use bodily states to rapidly modify choice selection rather than an inability to inhibit a previously rewarded response. The data are in agreement with theories advanced by several groups (Colwill and Rescorla, 1985; Balleine and Dickinson, 1998) indicating that, in the absence of the highly adaptable goal-directed behavior supported by areas 11 and 13 of the OFC, animals with early OFC damage are left with only an intact “habit” system to guide behavior. Thus, these animals will keep choosing items associated with previously positive outcomes rather than basing their choice on the current motivational value.

### Aversive behavioral flexibility

#### Baseline acoustic startle

All animals in groups showed an increase startle responses to increased noise intensity, although animals with neonatal OFC damage did show slightly, but not significantly, lower startle amplitudes across all intensities. However, it is possible that this group difference would have reached significance if the two Neo-C animals that had very high startle amplitudes outside the range of the measurement system were included in the control group. Overall, these findings parallel the lack of effects of selective ventromedial prefrontal lesions on baseline acoustic startle in rodents (Sullivan and Gratton, 2002).

#### Fear learning

Neonatal damage to OFC areas 11 and 13 also spared fear learning abilities. All animals regardless of group learned to associate the A+ cue with the aversive air puff with very little training. The normal fear learning after lesions of the prefrontal cortex is also consistent with rodent data (for review, see Sotres-Bayon and Quirk, 2010), but contrast with the fear conditioning deficits found after ventromedial prefrontal cortex damage in humans (Bechara et al., 1999), or after more generalized frontal-temporal damage as a result of Frontal-Temporal Dementia (Hoefer et al., 2008). Given that the OFC damage in human patients included prefrontal areas lying close to the middle line, which were not included in our study, it is likely that the different outcomes could be accounted by damage to these more ventromedial orbital fields.

#### Safety signal learning

The data provided little evidence for a role of OFC areas 11 and 13 in safety signal learning. To date, this is the first study to examine the role of the monkey OFC in acquiring safety signals and the lack of impairment may have resulted from the timing of the lesions. It should be acknowledged that one Neo-Oasp animal did have to be dropped because its startle responses became extremely high in the presence of all cues in the AX+/BX− phase of training. This might have resulted because by that time the animal became afraid of all cues, perhaps indicative of an inability to inhibit fear on the BX− trials. Although this proposal will await investigation of adult-onset OFC lesions on AX+/BX− task, an earlier study in rodents has shown that selective adult-onset damage to the ventral prefrontal cortex does not disrupt safety-signal learning (Gewirtz et al., 1997), whereas other structures such as the insula, anterior cingulate cortex, or striatum may be more relevant to safety-signal processing (Christianson et al., 2008, 2011; Kong et al., 2014). Given convincing evidence suggesting that fear learning is amygdala-dependent (Davis, 1992; Ledoux, 2000), whereas basic learning of appetitive associations are dependent on the striatum and ventromedial prefrontal cortex (Schiller et al., 2008), it is perhaps not too surprising that OFC areas 11 and 13 are not critical for safety signal learning. Indeed, using a fear conditioning reversal paradigm in humans, Schiller et al. (2008) paired one cue with a mild shock, while a second cue was paired with safety (no shock). Upon reversal of the reinforcement contingencies, neural activity shifted from the amygdala for the fearful cue to areas of the ventromedial prefrontal cortex and striatum as the cue now became associated with safety (Schiller et al., 2008). More importantly, there was an absence of neural activity modulation in the lateral sensory/orbital network during both contingencies. Thus, the present results support the human neuroimaging in positing that damage to the ventromedial OFC network may cause deficits in safety signal processing, whereas damage to the lateral orbital network is more disruptive to reward processing, and possibly higher order emotion-related behaviors (but see Gewirtz et al., 1997). This functional dissociation between the medial and lateral sectors of the OFC has recently been tested in monkeys (Noonan et al., 1999; Rudebeck and Murray, 2011) and is consistent with neuroanatomical findings indicating that the ventromedial OFC send more projections to the amygdala than it receives, whereas the lateral OFC receives more projections from the amygdala than it sends (Barbas, 2007). Thus, ventromedial OFC may be in a better position to regulate amygdala activity and this information might then be sent to the lateral OFC for further higher-order processing.

#### Flexible modulation of fear during conditioned inhibition

Just as we found no evidence for a lateral orbital network involvement in fear or safety-signal learning, there was little evidence that this lateral orbital network contributed to fear modulation. Both animals with neonatal OFC lesions and the sham-operated controls exhibited high fear-potentiated startle in the presence of the aversive A cue, low startle in the presence of the safety cue (B), and importantly intermediate startle when for the first time, the two cues were presented together (AB). Although Group Neo-Oasp did have a relatively lower fear-potentiated startle to the AX cue during the probe test than Group Neo-C, this group difference did not reach significance. The lower fear-potentiated startle in Group Neo-Oasp was largely driven by one case (see Table 5, Neo-Oasp-1) that startled less to the AX cue, than to the safety cue (B). Although Case Neo-Oasp-1 did have relatively more unintended damage to area 12 (see Table 1), a Pearson correlation matrix did not reveal any significant interactions between lesion extent of the various sub-regions of the OFC (both intended and unintended) and the ability to modulate fear-potentiated startle (all *ps* > 0.05).

#### Flexible modulation of fear during extinction

There was also no evidence of impaired ability to extinguish to the aversive cues (A−, AX−) after Neo-Oasp damage. These findings complement appetitive-related findings wherein both early and late selective damage to the lateral sensory/orbital network resulted in a sparing of reversal learning abilities (Kazama and Bachevalier, 2012), indicating that these animals are able to inhibit responses to cues that have become unrewarded. Again, this sparing contrasts with the severe flexible decision-making deficits that the same animals with Neo-Oasp lesions demonstrated in the Reinforcer Devaluation paradigm (see above). As compared to studies in rodents and humans, which often use aversive conditioning to study extinction, most of the studies on the role of the OFC in extinction and behavioral inhibition in nonhuman primates have generally used appetitive tasks, such as extinction of instrumental responses (Izquierdo and Murray, 2005) or object reversal (Jones and Mishkin, 1972) and go/nogo tasks (Swick et al., 2008). Thus, the lack of impairment following OFC lesions in fear extinction contrasts with the deficits observed in the extinction of instrumental responses, and suggest that the lateral orbital network may be more critical for the modulation of goal-actions associated with rewards than the regulation of fearful or anxious behaviors.

An alternative explanation for a lack of effects of Neo-Oasp on modulation of fear responses is that animals sustaining damage to areas 11 and 13 of the OFC in infancy were able to compensate by engaging other brain areas not normally mediating fear/safety-signal learning and fear modulation (Kennard, 1936; Goldman, 1976). We believe that this alternative explanation is unlikely given that the same animals with Neo-Oasp lesions showed severe impairment in negative emotion regulation under other circumstances. Thus, as compared to sham-operated controls, they displayed blunted fear reactivity to fearful stimuli as assessed by the Approach/Avoidance Paradigm (Raper et al., 2009) and did not modulate their behavioral reactivity according to levels of threat provided by a human intruder (Bachevalier et al., 2011). Thus, the evidence suggests that the lateral OFC network may not be required for the modulation or the extinction of basic fear responses but is rather implicated in fear modulation in situations involving higher-order processing, such as during perception and evaluation of complex or ambiguous social signals. Future studies will need to assess whether the same outcomes will follow damage to the lateral OFC network in adult monkeys. In addition, given that in humans and rodents, the lateral prefrontal areas 12 and ventromedial prefrontal areas 14 and 25 appear to be critical for both appetitive and aversive extinction (for review see Barbas, 2007; Price, 2007), studies assessing the effects of selective damage to these orbital frontal subfields on both conditioned inhibition and extinction processes may increase knowledge on the role of the different orbital frontal subfields in behavioral regulation.

### Comparisons with neonatal amygdala damage

As we stated in the introduction, the OFC critically interacts with the amygdala in support of flexible behavioral modulation (see Murray and Wise, 2010, for review). It is thus interesting to note that the current results on the effects of neonatal orbital frontal lesions on both the Reinforcer Devaluation task and the AX−/BX− task as well as those previously obtained on the same animals with Human Intruder paradigm (Raper et al., 2012) parallel remarkably with those obtained on the same three tasks in monkeys that had received neonatal damage to the amygdala (Bachevalier et al., 2011; Kazama et al., 2012; Kazama and Bachevalier, 2013; Raper et al., 2013). Thus, both types of neonatal lesions resulted in profound impairment in the modulation of behavioral responses based on the positive reward value of objects in the Devaluation Task, despite normal modulation of fear signals by safety signals in the AX+/BX− task. The only exceptions were the slight retardation in learning stimulus-reward association found after the neonatal OFC lesions but not the neonatal amygdala lesions and the slight retardation in conditioning to fear stimuli found after the neonatal amygdala lesions but not the neonatal OFC lesions. Thus, the two lesions may reflect different involvement of the OFC in the acquisition of stimulus-reward associations and of the amygdala in stimulus-fear conditioning. Given that the effects of both neonatal lesions on these two types of learning were very modest, these results will need to be replicated with larger sample sizes. In addition, both types of neonatal lesions impacted the abilities to regulate emotional reactivity after rapid changes in threatening social signals in the Human Intruder task. Interestingly, although the lesions of the OFC and of the amygdala were incurred in infancy at a time of significant brain plasticity, no other brain regions could compensate for the early loss of these brain structures. Altogether, the data suggest that interaction between OFC areas 11/13 and the amygdala play a critical role in the development of behavioral adaptation; an ability essential for the self-regulation of emotion and behavior that assures the maintenance of successful social relationships. This conclusion is further supported by human data indicating that early damage to the ventromedial portion of the prefrontal cortex in children is associated with impaired social and moral behavior (Anderson et al., 1999; Sánchez-Navarro et al., 2013) that could likewise have resulted from a lack of interactions between the orbital frontal cortex and the amygdala.

### Conflict of interest statement

The authors declare that the research was conducted in the absence of any commercial or financial relationships that could be construed as a potential conflict of interest.

## References

[B1] Anderson S. W. Bechara A. Damasio H. Tranel D. Damasio A. R. (1999). Impairment of social and moral behavior related to early damage in human prefrontal cortex. Nat. Neurosci. 2, 1032–1037 10.1038/12194 10526345

[B2] Antoniadis E. A. Winslow J. T. Davis M. Amaral D. G. (2007). Role of the primate amygdala in fear-potentiated startle: effects of chronic lesions in the rhesus monkey. J. Neurosci. 27, 7386–7396 10.1523/JNEUROSCI.5643-06.2007 17626199 PMC6672623

[B3] Bachevalier J. Machado C. J. Kazama A. (2011). Behavioral outcomes of late-onset or early-onset orbital frontal cortex (areas 11/13) lesions in rhesus monkeys. Ann. N.Y. Acad. Sci. 1239, 71–86 10.1111/j.1749-6632.2011.06211.x 22145877 PMC3740330

[B4] Balleine B. W. Dickinson A. (1998). Goal-directed instrumental action: contingency and incentive learning and their cortical substrates. Neuropharmacology 37, 407–419 10.1016/S0028-3908(98)00033-1 9704982

[B5] Barbaro J. Dissanayake C. (2007). A comparative study of the use and understanding of self-presentational display rules in children with high functioning autism and Asperger's disorder. J. Autism. Dev. Disord. 37, 1235–1246 10.1007/s10803-006-0267-y 17086441

[B6] Barbas H. (2000). Connections underlying the synthesis of cognition, memory, and emotion in primate prefrontal cortices. Brain Res. Bull. 52, 319–330 10.1016/S0361-9230(99)00245-2 10922509

[B7] Barbas H. (2007). Flow of information for emotions through temporal and orbitofrontal pathways. J. Anat. 211, 237–249 10.1111/j.1469-7580.2007.00777.x 17635630 PMC2375774

[B8] Barbas H. Henion T. H. Dermon C. R. (1991). Diverse thalamic projections to the prefrontal cortex in the rhesus monkey. J. Comp. Neurol. 313, 65–94 10.1002/cne.903130106 1761756

[B9] Baxter M. G. Parker A. Lindner C. C. Izquierdo A. D. Murray E. A. (2000). Control of response selection by reinforcer value requires interaction of amygdala and orbital prefrontal cortex. J. Neurosci. 20, 4311–4319 Available online at: http://www.jneurosci.org/content/20/11/4311.full.pdf+html 10818166 10.1523/JNEUROSCI.20-11-04311.2000 PMC6772657

[B10] Bechara A. Damasio H. Damasio A. R. Lee G. P. (1999). Different contributions of the human amygdala and ventromedial prefrontal cortex to decision-making. J. Neurosci. 19, 5473–5481 10377356 10.1523/JNEUROSCI.19-13-05473.1999 PMC6782338

[B10a] Brodmann K. (1909). Vergleichende Lokalisationslehre der Grosshirnrinde in ihren Prinzipien dargestellt auf Grund des Zellenbaues. Leipzig: Barth

[B11] Carmichael S. T. Price J. L. (1994) Architectonic subdivision of the orbital and medial prefrontal cortex in the macaque monkey. J. Comp. Neurol. 346, 366–402 10.1002/cne.903460305 7527805

[B11a] Christianson J. P. Benison A. M. Jennings J. Sandsmark E. K. Amat J. Kaufman R. D. (2008). The sensory insular cortex mediates the stress-buffering effects of safety signals but not behavioral control. J. Neurosci. 28, 13703–13711 10.1523/JNEUROSCI.4270-08.2008 19074043 PMC2667691

[B11b] Christianson J. P. Jennings J. H. Ragole T. Flyer J. G. Benison A. M. Barth D. S. (2011). Safety signals mitigate the consequences of uncontrollable stress via a circuit involving the sensory insular cortex and bed nucleus of the stria terminalis. Biol. Psychiatry 70, 458–464 10.1016/j.biopsych.2011.04.004 21684526 PMC3159417

[B12] Colwill R. M. Rescorla R. A. (1985). Instrumental responding remains sensitive to reinforcer devaluation after extensive training. J. Exp. Psych. 11, 520–536 10.1037/0097-7403.11.4.520

[B13] Davis M. (1992). The role of the amygdala in fear and anxiety. Annu. Rev. Neurosci. 15, 353–375 10.1146/annurev.ne.15.030192.002033 1575447

[B14] Del Casale A. Ferracuti S. Rapinesi C. Serata D. Piccirilli M. Savoja V. (2012). Functional neuroimaging in specific phobia. Psychiat. Res. 202, 181–197 10.1016/j.pscychresns.2011.10.009 22804970

[B15] Gallyas F. (1979). Silver staining of myelin by means of physical development. Neurol Res. 1, 203–209 95356 10.1080/01616412.1979.11739553

[B16] Gewirtz J. C. Falls W. A. Davis M. (1997). Normal conditioned inhibition and extinction of freezing and fear-potentiated startle following electrolytic lesions of medical prefrontal cortex in rats. Behav. Neurosci. 111, 712–726 10.1037/0735-7044.111.4.712 9267649

[B17] Goldman P. S. (1976). The role of experience in recovery of function following orbital prefrontal lesions in infant monkeys. Neuropsychologia 14, 401–412 10.1016/0028-3932(76)90069-5 825789

[B18] Goldman-Rakic P. S. Porrino L. J. (1985). The primate mediodorsal (MD) nucleus and its projection to the frontal lobe. J. Comp. Neurol. 242, 535–560 10.1002/cne.902420406 2418080

[B19] Gottfried J. A. O'Doherty J. Dolan R. J. (2003). Encoding predictive reward value in human amygdala and orbitofrontal cortex. Science 301, 1104–1107 10.1126/science.1087919 12934011

[B20] Goursaud A. P. Bachevalier J. (2007). Social attachment in juvenile monkeys with neonatal lesion of the hippocampus, amygdala and orbital frontal cortex. Behav. Brain Res. 176, 75–93 10.1016/j.bbr.2006.09.020 17084912

[B21] Grillon C. Ameli R. (2001). Conditioned inhibition of fear-potentiated startle and skin conductance in humans. Psychophysiology 38, 807–815 10.1111/1469-8986.3850807 11577904

[B22] Heuer E. Kazama A. M. Davis M. Bachevalier J. (2010). Prepulse inhibition following selective neonatal lesions of the amygdala, hippocampus or orbital frontal cortex in the rhesus monkey, in Poster Presentation at Society for Neuroscience Meeting, (San Diego, CA).

[B23] Hodos W. Bobko P. (1984). A weighted index of bilateral brain lesions. J. Neurosci. Methods 12, 43–47 10.1016/0165-0270(84)90046-3 6513590

[B24] Hoefer M. Allison S. C. Schauer G. F. Neuhaus J. M. Hall J. Dang J. N. (2008). Fear conditioning in frontotemporal lobar degeneration and Alzheimer's disease. Brain 131, 1646–1657 10.1093/brain/awn082 18492729 PMC2544622

[B25] Izquierdo A. Murray E. A. (2005). Opposing effects of amygdala and orbital prefrontal cortex lesions on the extinction of instrumental responding in macaque monkeys. Eur. J. Neurosci. 22, 2341–2346 10.1111/j.1460-9568.2005.04434.x 16262672

[B26] Izquierdo A. Murray E. A. (2007). Selective bilateral amygdala lesions in rhesus monkeys fail to disrupt object reversal learning. J. Neurosci. 27, 1054–1062 10.1523/JNEUROSCI.3616-06.2007 17267559 PMC6673199

[B27] Izquierdo A. Suda R. K. Murray E. A. (2004). Bilateral orbital prefrontal cortex lesions in rhesus monkeys disrupt choices guided by both reward value and reward contingency. J. Neurosci. 24, 7540–7548 10.1523/JNEUROSCI.1921-04.2004 15329401 PMC6729636

[B28] Jones B. Mishkin M. (1972). Limbic lesions and the problem of stimulus–reinforcement associations. Exp. Neurol. 36, 362–377 10.1016/0014-4886(72)90030-1 4626489

[B29] Jovanovic T. Kazama A. Bachevalier J. Davis M. (2012). Impaired safety signal learning may be a biomarker of PTSD. Neuropharmacology 62, 695–704 10.1016/j.neuropharm.2011.02.023 21377482 PMC3146576

[B30] Kazama A. Bachevalier J. (2009). Selective aspiration or neurotoxic lesions of orbital frontal areas 11 and 13 spared monkeys' performance on the object discrimination reversal task. J. Neurosci. 29, 2794–2804 10.1523/JNEUROSCI.4655-08.2009 19261875 PMC2701144

[B31] Kazama A. M. Bachevalier J. (2012). Preserved stimulus-reward and reversal learning after selective neonatal orbital frontal areas 11/13 or amygdala lesions in monkeys. Dev. Cogn. Neurosci. 2, 363–380 10.1016/j.dcn.2012.03.002 22494813 PMC3369024

[B32] Kazama A. M. Bachevalier J. (2013). Effects of selective neonatal amygdala damage on concurrent discrimination learning and reinforcer devaluation in monkeys. J. Psychol. Psychother. S7:005 10.4172/2161-0487.S7-005 24567865 PMC3932052

[B33] Kazama A. M. Glavis-Bloom C. Bachevalier J. (2008). Neonatal amygdala and orbital frontal cortex lesions disrupt flexible decision-making in adult macaques, in Poster Presentation at Society for Neuroscience Meeting, (Washington, DC).

[B34] Kazama A. M. Heuer E. Davis M. Bachevalier J. (2010). Long-term effects of selective neonatal lesions of the amygdala, hippocampus, or areas 11 and 13 of the orbitofrontal cortex on fear regulation, in Poster Presentation at Society for Neuroscience Meeting, (San Diego, CA).

[B35] Kazama A. M. Heuer E. Davis M. Bachevalier J. (2012). Effects of neonatal amygdala lesions on fear learning, conditioned inhibition, and extinction in adult macaques. Behav. Neurosci. 126, 392–403 10.1037/a0028241 22642884 PMC3740331

[B37] Kennard M. A. (1936). Age and other factors in motor recovery from precentral lesions in monkeys. Am. J. Physiol. 115, 138–146

[B11c] Kong E. Monje F. J. Hirsch J. Pollak D. D. (2014). Learning not to fear: neural correlates of learned safety. Neuropsychopharmacology 39, 515–527 10.1038/npp.2013.191 23963118 PMC3895233

[B38] Ledoux J. E. (2000). Emotion circuits in the brain. Annu. Rev. Neurosci. 23, 155–184 10.1146/annurev.neuro.23.1.155 10845062

[B39] Machado C. J. Bachevalier J. (2006). The impact of selective amygdala, orbital frontal cortex, or hippocampal formation lesions on established social relationships in rhesus monkeys (*Macaca mulatta*). Behav. Neurosci. 120, 761–786 10.1037/0735-7044.120.4.761 16893284

[B40] Machado C. J. Bachevalier J. (2007a). The effects of selective amygdala, orbital frontal cortex or hippocampal formation lesions on reward assessment in nonhuman primates. Eur. J. Neurosci. 25, 2885–2904 10.1111/j.1460-9568.2007.05525.x 17561849

[B41] Machado C. J. Bachevalier J. (2007b). Measuring reward assessment in a semi-naturalistic context: the effects of selective amygdala, orbital frontal or hippocampal lesions. Neuroscience 148, 599–611 10.1016/j.neuroscience.2007.06.035 17693034 PMC2064940

[B42] Machado C. J. Kazama A. M. Bachevalier J. (2009). Impact of amygdala, orbital frontal, or hippocampal lesions on threat avoidance and emotional reactivity in nonhuman primates. Emotion 9, 147–163 10.1037/a0014539 19348528 PMC4410686

[B43] Malkova L. Gaffan D. Murray E. A. (1997). Excitotoxic lesions of the amygdala fail to produce impairment in visual learning for auditory secondary reinforcement but interfere with reinforcer devaluation effects in rhesus monkeys. J. Neurosci. 17, 6011–6020 9221797 10.1523/JNEUROSCI.17-15-06011.1997 PMC6573210

[B44] Meunier M. Bachevalier J. Mishkin M. (1997). Effects of orbital frontal and anterior cingulate lesions on object and spatial memory in rhesus monkeys. Neuropsychologia 35, 999–1015 10.1016/S0028-3932(97)00027-4 9226661

[B45] Morecraft R. J. Geula C. Mesulam M. M. (1992). Cytoarchitecture and neural afferents of orbitofrontal cortex in the brain of the monkey. J. Comp. Neurol. 323, 341–358 10.1002/cne.903230304 1460107

[B46] Murray E. A. Wise S. P. (2010). Interactions between orbital prefrontal cortex and amygdala: advanced cognition, learned responses and instinctive behaviors. Curr. Opin. Neurobiol. 20, 212–220 10.1016/j.conb.2010.02.001 20181474 PMC2862864

[B47] Myers K. M. Davis M. (2004). AX+, BX- discrimination learning in the fear-potentiated startle paradigm: possible relevance to inhibitory fear learning in extinction. Learn. Mem. 11, 464–475 10.1101/lm.74704 15254216 PMC498334

[B48] Noonan M. P. Walton M. E. Behrens T. E. J. Sallet J. Buckley M. J. Rushworth M. F. S. (1999). Separate value comparison and learning mechanisms in macaque medial and lateral orbitofrontal cortex. Proc. Natl. Acad. Sci. U.S.A. 107, 20547–20552 10.1073/pnas.1012246107 21059901 PMC2996698

[B49] O'Doherty J. Kringelbach M. L. Rolls E. T. Hornak J. Andrews C. (2001). Abstract reward and punishment representations in the human orbitofrontal cortex. Nat. Neurosci. 4, 95–102 10.1038/82959 11135651

[B51] Ongur D. Price J. L. (2000). The organization of networks within the orbital and medial prefrontal cortex of rats, monkeys and humans. Cereb. Cortex 10, 206–219 10.1093/cercor/10.3.206 10731217

[B52] Payne C. Goursaud A.-P. Kazama A. M. Bachevalier J. (2007). The effects of neonatal amygdala and orbital frontal lesions on the development of dyadic social interactions in infant rhesus monkeys, Poster Presentation at Society for Neuroscience Meeting, (San Diego, CA).

[B53] Pickens C. L. Saddoris M. P. Setlow B. Gallagher M. Holland P. C. Schoenbaum G. (2003). Different roles for orbitofrontal cortex and basolateral amygdala in a reinforcer devaluation task. J. Neurosci. 23, 11078–11084 Available online at: http://www.jneurosci.org/content/23/35/11078.full.pdf+html 14657165 10.1523/JNEUROSCI.23-35-11078.2003 PMC6741041

[B53b] Pixley G. L. Malkova L. Webster M. J. Mishkin M. Bachevalier J. (1997). Early damage to both inferior convexity and orbital prefrontal cortices impairs DNMS learning in infant monkeys. Abstr. Soc. Neurosci. 23

[B53a] Price J. L. (2007). Definition of the orbital cortex in relation to specific connections with limbic and visceral structures and other cortical regions. Ann. N.Y. Acad. Sci. 1121, 54–71 10.1196/annals.1401.008 17698999

[B54] Raper J. R. Kazama A. M. Bachevalier J. (2009). Blunted fear reactivity after neonatal amygdala and orbital frontal lesions in rhesus monkeys. Poster Presentation at Society for Neuroscience Meeting, (Chicago, IL).

[B55] Raper J. Wilson M. Sanchez M. Machado C. J. Bachevalier J. (2013). Pervasive alterations of emotional and neuroendocrine responses to an acute stressor after neonatal amygdala lesions in rhesus monkeys. Psychoneuroendocrinology 38, 1021–1035 10.1016/j.psyneuen.2012.10.008 23148887 PMC3593974

[B56] Raper J. R. Wilson M. Sanchez M. Bachevalier J. (2012). Neonatal orbital frontal damage alters basal cortisol and emotional reactivity, but not stress reactive cortisol response, in adult rhesus monkeys. International Society for Psychoneuroendocrinology Meeting, 2012, Euro. J. Psychotraumatol. 3 supp1., 100

[B57] Ray J. P. Price J. L. (1993). The organization of projections from the mediodorsal nucleus of the thalamus to orbital and medial prefrontal cortex in macaque monkeys. J. Comp. Neurol. 337, 1–31 10.1002/cne.903370102 7506270

[B58] Reed P. Watts H. Truzoli R. (2013). Flexibility in young people with autism spectrum disorders on a card sort task. Autism 17, 162–171 10.1177/1362361311409599 21690212

[B59] Rudebeck P. H. Murray E. A. (2011). Dissociable effects of subtotal lesions within the macaque orbital prefrontal cortex on reward-guided behavior. J. Neurosci. 31, 10569–10578 10.1523/JNEUROSCI.0091-11.2011 21775601 PMC3171204

[B60] Sánchez-Navarro J. P. Driscoll D. Anderson S. W. Tranel D. Bechara A. Buchanan T. W. (2013). Alterations of attention and emotional processing following childhood-onset damage to the prefrontal cortex. Behav. Neurosci. 128, 1–11 10.1037/a0035415 24377423 PMC4324722

[B61] Schiller D. Levy I. Niv Y. Ledoux J. E. Phelps E. A. (2008). From fear to safety and back: reversal of fear in the human brain. J. Neurosci. 28, 11517–11525 10.1523/JNEUROSCI.2265-08.2008 18987188 PMC3844784

[B62] Shepherd A. M. Laurens K. R. Matheson S. L. Carr V. J. Green M. J. (2012). Systematic meta-review and quality assessment of the structural brain alterations in schizophrenia. Neurosci. Biobehav. Rev. 36, 1342–1356 10.1016/j.neubiorev.2011.12.015 22244985

[B63] Shin L. M. Rauch S. L. Pitman R. K. (2006). Amygdala, medial prefrontal cortex, and hippocampal function in PTSD. Ann. N.Y. Acad. Sci. 1071, 67–79 10.1196/annals.1364.007 16891563

[B64] Sotres-Bayon F. Quirk G. J. (2010). Prefrontal control of fear: more than just extinction. Curr. Opin. Neurobiol. 20, 231–235 10.1016/j.conb.2010.02.005 20303254 PMC2878722

[B65] Sullivan R. M. Gratton A. (2002). Behavioral effects of excitotoxic lesions of ventral medial prefrontal cortex in the rat are hemisphere-dependent. Brain Res. 927, 69–79 10.1016/S0006-8993(01)03328-5 11814433

[B66] Swick D. Ashley V. Turken A. U. (2008). Left inferior frontal gyrus is critical for response inhibition. BMC Neurosci. 9:102 10.1186/1471-2202-9-102 18939997 PMC2588614

[B67] West E. A. Forcelli P. A. Murnen A. T. Mccue D. L. Gale K. Malkova L. (2012). Transient inactivation of basolateral amygdala during selective satiation disrupts reinforcer devaluation in rats. Behav. Neurosci. 126, 563–574 10.1037/a0029080 22845705 PMC3432320

[B68] Winslow J. T. Noble P. L. Davis M. (2008). AX+/BX- discrimination learning in the fear-potentiated startle paradigm in monkeys. Learn Mem. 15, 63–66 10.1101/lm.843308 18230674

[B69] Winslow J. T. Parr L. A. Davis M. (2002). Acoustic startle, prepulse inhibition, and fear-potentiated startle measured in rhesus monkeys. Biol. Psychiatry 51, 859–866 10.1016/S0006-3223(02)01345-8 12022958

[B70] Zeeb F. D. Winstanley C. A. (2013). Functional disconnection of the orbitofrontal cortex and basolateral amygdala impairs acquisition of a rat gambling task and disrupts animals' ability to alter decision-making behavior after reinforcer devaluation. J. Neurosci. 33, 6434–6443 10.1523/JNEUROSCI.3971-12.2013 23575841 PMC6619089

